# Effect of 0.8mg/ml Losartan on Corneal Opacities

**DOI:** 10.12669/pjms.41.3.11237

**Published:** 2025-03

**Authors:** Aisha Fawad, Waqar Muzaffar, Unnab Anjum, Mahad Naseer Amer

**Affiliations:** 1 Aisha Fawad, FCPS (Ophth), FRCSGlas (Ophth) Associate Professor of Ophthalmology, Refractive Department, National University of Medical Sciences, National University of Medical Sciences, Rawalpindi, Pakistan; 2 Waqar Muzaffar, FCPS (Ophth), FRCS (Edinburgh), CST (UK) Professor and Dean of Ophthalmology, National University of Medical Sciences, National University of Medical Sciences, Rawalpindi, Pakistan; 3 Unnab Anjum Resident Ophthalmology, National University of Medical Sciences, Rawalpindi, Pakistan; 4 Mahad Naseer Amer Resident Ophthalmology, National University of Medical Sciences, Rawalpindi, Pakistan

**Keywords:** Losartan, Corneal opacity, keratitis

## Abstract

Losartan, commonly used as an angiotensin receptor II inhibitor for hypertension, has recently revealed additional therapeutic properties. Studies demonstrate its potential in treating liver fibrosis, mitigating heart disease secondary to radiation, reducing skin scar formation, and even reversing renal cortical fibrosis. In a specific case, a female patient suffering from persistent bilateral nummular keratitis and corneal opacities experienced significant improvement in her left eye after using 0.8mg/ml Losartan eye drops. Untreated right eye showed no change. Based on the positive outcome, patient was offered same treatment in her right eye as well with monthly follow-ups, which she happily accepted. The stark contrast between the treated and untreated eye highlights the promising role of Losartan in treating corneal opacities.

## INTRODUCTION

A commonly used anti-hypertensive agent, Losartan which is classified as an angiotensin receptor II inhibitor, has long been used in humans with well-known safety profile and side effects.^1^ Losartan has also been shown in recent studies to have TGF b inhibiting properties.^2^ It is precisely due to this property that enabled us to explore newer horizons and see what this drug may offer. For instance, human trials conducted to ascertain inhibitory action of Losartan on liver fibrosis has shown promising results.^3^ Moreover, a recently orchestrated animal trial on heart disease secondary to radiation has demonstrated similar positive effects.^2^

In addition to this, Losartan’s fibrinolytic qualities have proven to reduce scar formation on skin.^4^ In the field of ophthalmology animal trials have shown topical Losartan to reduce corneal stromal opacities.^5^,^6^ Systemic drug does not penetrate human cornea and human case reports have been done to show that no significant side effects have occurred on human subjects with topical Losartan drops.^7^ Currently corneal opacities are either treated conservatively with topical anti-inflammatory drops or surgically with laser phototherapeutic keratectomy (PTK) and keratoplasty. Addition of a topical drop like Losartan that can treat corneal opacities satisfactorily will contribute hugely towards ophthalmic patient care.

## CASE PRESENTATION

Our patient was an eighteen-year-aged female who presented to eye outpatient department of Tertiary Care Public Sector Eye Hospital, Rawalpindi with persistent corneal opacities following post Adenoviral nummular keratitis for the last two years. She had opacities in anterior stroma which had been persistent for over two years and was resistant to previous treatment regimen, including topical steroids, lubricants and cyclosporine drops. Her uncorrected visual acuity (UCVA) was 6/20 in right eye and 6/30 in left eye, best corrected visual acuity (BCVA) was 6/12 in right eye and 6/15 in left eye. She was referred to Refractive Department for an opinion regarding PTK (Photo therapeutic keratectomy) since the opacities appeared to be dense, located in the anterior stroma and visually significant. On anterior segment optical coherence tomography (OCT), her opacities were at a depth of 100 to 180 microns. Due to thin corneas, (pachymetry of 469 and 471 microns in right and left eye respectively) she was not considered suitable for the laser procedure and was kept on medical therapy. Her fundus findings were unremarkable in both eyes with media clear and no fundus pathologies, IOP was 12 in right eye and 13 in left eye. She was given a trial of topical Losartan, after taking a detailed informed consent. Whole process was thoroughly explained to her and was advised to have regular four weekly follow ups.

**Fig.1:**
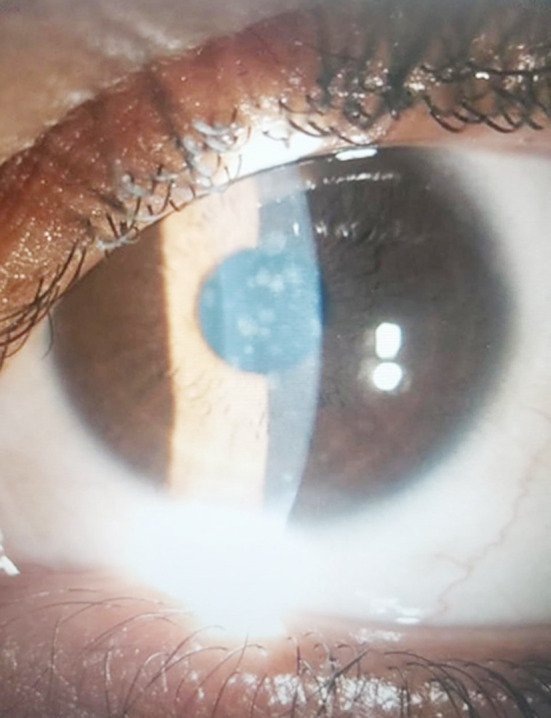
Left eye- pretreatment

**Fig.2:**
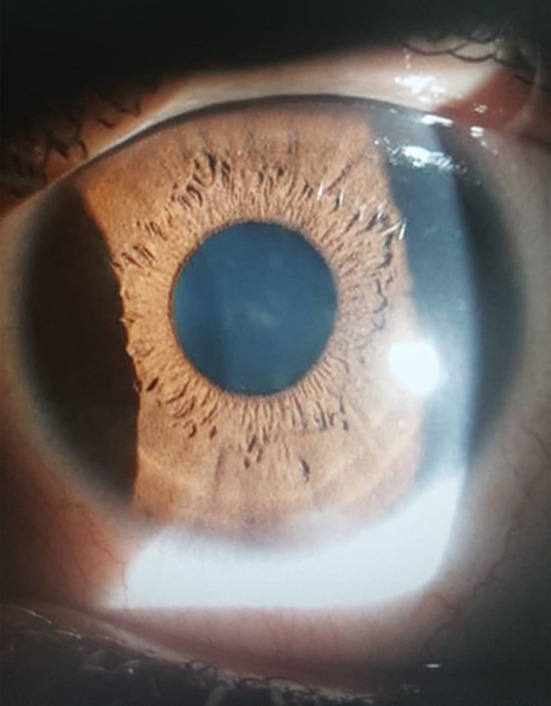
Left eye -post treatment.

She was started on a regimen of 0.8mg/ml Losartan, which was prepared by crushing Losartan tablets and diluting in sterile balanced electrolyte solution in a sterile setup, six times daily in her left eye (the eye with denser opacities). Dose concentration and frequency of drops was derived from guidelines as given by Wilson SE.^6^ In her right eye she was advised to continue her standard treatment which comprised of steroids and lubricants. She was asked to follow up weekly and she was also given a phone line to contact immediately in case she experienced any problems. A month later, she showed significant improvement in opacities on the left side. Her Losartan treated cornea showed only faint presence of the past opacities while the condition of her right eye remained unchanged (Fig.1-2). Her BCVA one month later was 6/9 in left eye whereas she remained at 6/12 in her right eye. Her feedback was negative for any side effects, intra ocular pressure were 14 in the right and 14 in the left, hence was offered the same treatment in the other eye. Further down the lane, her right eye followed the same pattern of improvement in the density of her opacities. Patient remained stable after four months of follow up and did not report any ocular or systemic side effects during this time period. We intend to slowly taper her Losartan drops over a period of next two months to assess what the effect of reduced dosage would be on the opacities and keep her in follow up for any recurrence.

## DISCUSSION

The effect Losartan had on the patient’s left eye was very promising and the contrast provided with the right eye on which this regimen was not started was indeed stark, further pointing towards the role of Losartan eye drops in resolving the said opacities. The patient had no complaints upon the continuous use of these eye drops though more testing is needed to talk about side effects of this formulation with any amount of certainty, in this case at least the patient reported a great deal of satisfaction with the formulation and was eager to start using it on her other eye as well. A case report by Pereira-Souza AL et al used similar regimen and reported improvement in corneal opacity at four months, the initial cause of which was a complicated LASIK procedure in a female.^5^ Although these results are in line with the few isolated case reports that have already been published, many questions are yet to be answered. These results do warrant further trials of Losartan in more clinical scenarios specially its long-term effects on eye and general health.

### Authors Contribution

**AF** conceived, designed and edited the manuscript, is responsible for integrity of research.

**MNA and UA** did data collection and manuscript writing along with the preparation and calculations of the medication.

**WM** did review and final approval of manuscript.
